# Combined effects of naringin and doxorubicin on the JAK/STAT signaling pathway reduce the development and spread of breast cancer cells

**DOI:** 10.1038/s41598-024-53320-9

**Published:** 2024-02-03

**Authors:** Heba Effat, Hamed A. Abosharaf, Aliaa M. Radwan

**Affiliations:** 1 Medical Biochemistry and Molecular Biology Unit, Department of Cancer Biology, National Cancer Institut, Cairo University, Cairo, Egypt; 2 Biochemistry Division, Chemistry Department, Faculty of Science, Tanta University, Tanta, Egypt

**Keywords:** Biochemistry, Cancer

## Abstract

Breast cancer therapy options are limited due to its late diagnosis and poor prognosis. Doxorubicin is the fundamental therapy approach for this disease. Because chemotherapy has numerous adverse effects, the scope of the existing research was to appraise the synergetic effect of doxorubicin and naringin and explore the underlying mechanism. The cytotoxicity of doxorubicin and naringin on MCF-7 was monitored. Furthermore, the expression of STAT3 and JAK1 as well as the apoptotic and metastatic related genes (Bax, Bcl-2, Survivin, and VEGF) were conducted by immunoblotting assay and qRT-PCR. In addition, a wound healing test was utilized to appraise the migration and metastasis of MCF-7. Our results revealed that naringin and doxorubicin had a synergetic inhibitory influence on MCF-7 cells growth and migration. The synergetic action of doxorubicin and naringin effectively hindered the expression of STAT3, JAK1, Bcl-2, Survivin, and VEGF, with a boost in the level of Bax compared to cells treated with either doxorubicin or naringin. In conclusion, our findings imply that combining doxorubicin with naringin may be a favorable strategy for inhibiting the growth of breast cancer.

## Introduction

Breast cancer is the top frequent and the major prevalent reason of death among Egyptian women. Despite significant progress in many developing countries, Egypt's 5-year survival rate remained lower, between 28 and 68%, as a result of late diagnosis and a insufficiently prognosis^1^. Breast cancer treatment is often complicated and may be ineffective due to intrinsic or acquired drug resistance in cancer cells and severe side effects of chemotherapeutic agents^2,3^. One of the most popular anticancer medications is doxorubicin, which is used to treat both metastatic and early-stage breast cancer. Unfortunately, because of its oxidative stress action, its use is linked to the emergence of severe cumulative dose-related cardiotoxicity, myelosuppression, and treatment resistance^4^. As a result, it is preferable to combine it with other anticancer agents in order to lower its dosage without sacrificing its effectiveness^5^.

Different research reported that high intake of vegetables and fruits may prevent cancer growth and decreased risk of different human cancers due to the presence of polyphenolic compounds, flavonoids^6–9^. Flavonoids are a big class of phenol-containing phytochemicals found in many fruits, vegetables, and some medicinal herbs that exert different biological functions involving antioxidant, anti-inflammatory, and anticancer^10,11^.

Naringin (4′,5,7-Trihydroxyflavanone-7-rhamnoglucoside) is natural flavonoid found in grapefruit, orange, and Kino^12,13^. Previous investigations have revealed that naringin has numerous pharmacological impacts, such as anti-inflammatory, antioxidant, antimicrobial, and anti-carcinogenic activities^14^. Furthermore, it has the capability to reduce cell propagation and growth in various human malignancies like lung, melanoma, and breast by targeting several signal transduction pathways^15,16^. Li et al., exhibited that naringin reduced cell outgrowth and propped up apoptosis as well as blocked cell cycle at G0/G1phase in triple negative breast cancer by suppressing the pathway of β-catenin^17^. Zhou et al., suggested that naringin restrained the growth of thyroid cancer cells and encouraged the apoptosis by suppressing the path of phosphatidylinositol 3-kinase/protein kinase B (PI3K/AKT)^18^.

The signaling pathway of Janus kinase/signal transducers and activators of transcription (JAK/STAT) constitute an important signaling machinery which regulate many critical cellular activities such as proliferation, development, and differentiation, inflammation, apoptosis, and angiogenesis^19^. It has been previously informed that abnormal activation of STAT3 induce oncogenic processes in various cancers such as prostate, lung, ovary, leukemia, and breast^20,21^. Therefore, finding an inhibitor of STAT3 might be a promising approach in prevention and targeted therapy of cancer. Thus, this study was designated to investigate the antitumor activity of naringin alone or combined with doxorubicin against breast cancer through blockage of the JAK/STAT signaling route.

## Methods

### Cell culture

Breast malignant cell line, MCF-7, and human skin fibroblast (HSF) from VACSERA, (the Egyptian Company for Production of Vaccines, Sera, and Drugs) were grown in RPMI-1640 medium with high glucose and L-glutamine content, 10% fetal bovine serum (FBS), and 1% streptomycin/penicillin (100 µg/ml streptomycin and 100 U/ml penicillin) (Sigma Aldrich Chemical Co., St. Louis, MO, USA). Cells were kept at 37 °C with 5% CO_2_. When confluent, they were passaged at 1:4 ratio.

### Cell viability assay

The cytotoxic impact of naringin (98% purity, Solarbio, China) and doxorubicin (Sigma, USA) on the growth of MCF-7 cells was evaluated using the previously reported Sulforhodamine B (SRB) assay (Sigma-Aldrich Chemical Co., USA)^22^. In addition, the cytotoxic effect of naringin on HSF cells was evaluated. In a 96-well plate, MCF-7, and HSF cells (4.0 × 10^3^ cells/well) were seeded. On the next day, MCF-7 cells were subjected to various dosages of naringin (NAR) (5, 10, 15, 20 and 25 μg/ml), doxorubicin (DOX) (1, 2, 4, 8, 16 and 32 μg/ml) for 48h. HSF cells were subjected to different NAR concentrations (5, 10, 15, 20, 25, 50, and 100 μg/ml). After treatments, cells were settled in 10% trichloroacetic acid for one hour at 4 °C. Finally, SRB 0.4% (w/v) was added for 30 min before washing with 1% acetic acid. SRB-bound cells have been dissolved down by a pH 10.5 solution of 10 mM Tris. Using a microplate reader, the absorbance was determined at 570 nm (TecanSunriseTM, Germany). Half-maximal inhibitory concentration (IC_50_) values, utilizing sigmoidal fitting equation, were calculated (GraphPad Prism program).

### Colony formation assay

The antiproliferative effect of doxorubicin and naringin on MCF-7 cells were confirmed using clonogenicity assay. In 6-well plate, 1 × 10^3^ MCF-7 cells were plated and treated for 48h with 1/2 IC_50_ DOX, IC_50_ NAR and their combination. The media was then replaced with fresh media and the cells were allowed to grow and proliferate for 10 days at 37 °C. After that, the colonies were fixed with methanol and stained with 0.5% crystal violet. The colonies of 50 cells were imaged and counted using inverted microscope^23^.

### Quantitative real-time polymerase chain reaction (qRT-PCR)

500,000 MCF-7 cells were planted in 25 mm flasks. After overnight incubation, cells were given 1/2 IC_50_ DOX, IC_50_ NAR and a combination (Comb) of 1/2 IC_50_ DOX and IC_50_ NAR for 48h. Using the miRNeasy Mini Kit (Qiagen, Germany), total RNA was purified from treated and untreated cells in accordance with the Qiagen company's guidelines. A NanoDrop-2000 spectrophotometer (ThermoFisher Scientific, USA) was used to measure the amount of RNA. Utilizing the Quantitect RNA reverse transcription kit (Qiagen, Germany) in accordance with the manufacturer's guidelines, complementary DNA (cDNA) was synthesized from 1 µg of RNA. qPCR was used to determine the relative expression levels of STAT3, JAK1, Bcl-2 associated x protein (Bax), B-cell lymphoma 2 (Bcl-2), Survivin, vascular endothelial growth factor (VEGF), matrix metalloproteinases (MMP-2, MMP-9) using a quantinova SYBR Green reagent kit from Qiagen with cDNA as the template. As an internal control for mRNAs, glyceraldehyde 3-phosphate dehydrogenase (GAPDH) was used. We bought primer assays from Qiagen in Germany. On the ViiA™ 7 PCR machine (Applied Biosystems, USA), all the qPCR reactions were run in triplicate. Data were analysed using the ΔΔCt comparative approach and the fold of change = 2^−ΔΔCt^^24^. The primers used in this work are ready made (Cat No. 249900, Qiagen, Germany). JAK1(GeneGlobe ID-QT00050225), STAT3 (GeneGlobe ID-QT00068754), Bax (GeneGlobe ID-QT00031192), Bcl-2 (GeneGlobe ID-QT00025011), Survivin (GeneGlobe ID-QT01679664), VEGF (GeneGlobe ID-QT01682072), MMP-2 (GeneGlobe ID-QT00088396), and MMP-9 (GeneGlobe ID-QT00040040).

### Western blot analysis

Naringin and doxorubicin-treated cells were assessed by western blot analysis for STAT3, p-STAT3, JAK1, p-JAK1, Bax, Bcl-2, Survivin, and VEGF proteins in whole-cell extracts as previously described^25^. The cells were lysed in an ice-cold lysis solution that contained the following ingredients: 50 mM Tris–HCl pH 7.4, 150 mM NaCl, 1% Nonidet P-40, 1 mM ethylenediaminetetraacetic acid (EDTA), and 1X Protease Inhibitor Cocktail to create the cell extracts. Using the Bradford Protein Assay Kit (SK3041) (Bio Basic Inc, Ontario, Canada), the protein concentration in each sample was calculated according to manufacturer's guidelines. After electrophoresis, the proteins were electrotransferred to a polyvinylidene fluoride (PVDF) membrane, blocked for 1 h at room temperature with 3% bovine serum albumin, then probed with a variety of primary antibodies (Table 1) overnight at 4 °C. The membrane was then washed, kept for 1 h at room temperature with a horseradish peroxidase (HRP)-conjugated secondary antibody solution, and lastly analyzed using an enhanced chemiluminescence (ECL) reagent. Original images (in supplementary file) were captured using UVITEC digital imaging system which typically produces highly contrast bands. These bands were then quantified by its software package (UK) and the densities of each band were normalized to β-actin.

**Table 1:** Antibodies used in western blot analysis.

Antibody	Dilution	Size (kDa)	Cat No.	Company
Anti-STAT3	1:1000	88	ab68153	Abcam
Anti-JAK1	1:1000	130	#3332	Cell Signaling
Anti-Bax	1:1000	23	SC-7480	Santa Cruz Biotechnology
Anti-Bcl-2	1:1000	26	SC-7382	Santa Cruz Biotechnology
Anti-survivin	1:1000	16	#2808	Cell Signaling
Anti-VEGF	1:1000	21	SC-7269	Santa Cruz Biotechnology
Anti phospho-STAT3	1:1000	86	#9131	Cell Signaling
Anti phospho-JAK1	1:1000	130	# PA5-104554	Thermo Fisher Scientific
Anti-β-actin	1:5000	43	**# **MA1-91399	Thermo Fisher Scientific

### Cell migration test

In 6-well plates, MCF-7 cells were cultivated to form confluent monolayers. The cells were then scraped with a sterile pipette in the center of the well to form a line. The cells were subsequently given a 48 h treatment of 1/2 IC_50_ DOX, IC_50_ NAR, and their mixture. ImageJ software was used to analyze photographs of cell migration into the scratched area at 0 h and 48 h after treatment to assess the healing effect.

### Apoptosis analysis

Flow cytometric analysis was utilized to measure the percentage of cells that undergo apoptosis using Annexin V detection kit (Immunostep, Spain*)* according to manufacturer's protocol. In brief, MCF-7 cells were resuspended in 1X Annexin-binding buffer after being collected and washed with PBS. This suspension was then mixed with Annexin-V/PI staining solution. After 15 min of dark incubation, the samples were analyzed using a Beckman Coulter, USA^26^. The gating strategy for annexin-V interpretation according to PI applied on dot plot forward side scatter (FSC) and side scatter (SSC) which represent the expression of stain fluorescence on cells.

### In silico molecular docking

The molecular docking program Autodock 1.5.6 (https://ccsb.scripps.edu/mgltools/downloads) was used to perform virtual screening and examine the biochemical interactions between naringin (https://pubchem.ncbi.nlm.nih.gov/compound/442428) and STAT3 protein. The macromolecule STAT3's crystal structure (ID code: 1BG1) was acquired from Protein Data Bank server (https://www.rcsb.org/structure/1BG1). Autodock 1.5.6 tool was used for protein and ligand preparation, the polar hydrogen and Gasteiger charges were embedded into the receptor protein then the PDBQT files for both ligand and protein were created by setting number of torsions for each. The receptor protein was encircled by a grid box with dimensions of 80 by 80 by 80 and a spacing of 0.375. The docking study was carried out using Lamarckian genetic algorithm (LGA) with default values. The best docked conformation was selected depending on the values of inhibition constant (ki, mM) and binding energy (kcal/mol). Then the results were analyzed and interpreted using Discovery Studio Visualizer (DSV) and PyMOL software^27,28^.

### Statistical calculations

GraphPad prism package software (CA, USA) was employed to estimate the statistical differences in the existing study using one-way ANOVA followed by post-hoc Tukey’s test. The data were presented as mean ± SD, n = 3, *p* ˂ 0.05 counted as significantly differences.

## Results

### Impact of doxorubicin and naringin on MCF-7 cells viability

**Figure 1:**
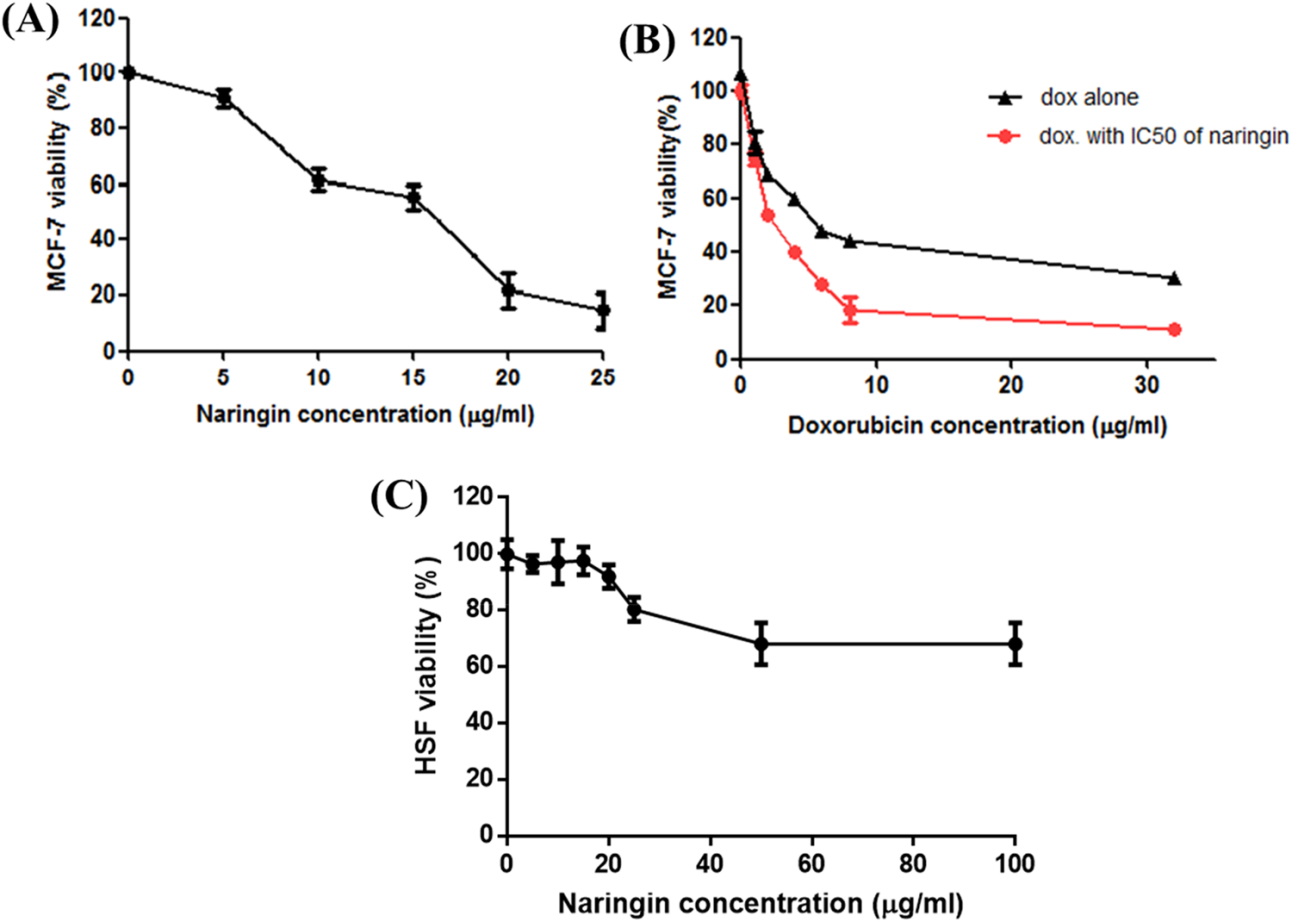
Impact of doxorubicin and naringin on cells viability. (**A,B**) MCF-7 cells were exposed to multiple dosages of doxorubicin and naringin. (**C**): cytotoxicity of naringin against HSF cells. Cell viability was determined by SRB assay after 48 h. Data were expressed as Mean ± SD of three experiments performed in triplicates.

**Figure 2:**
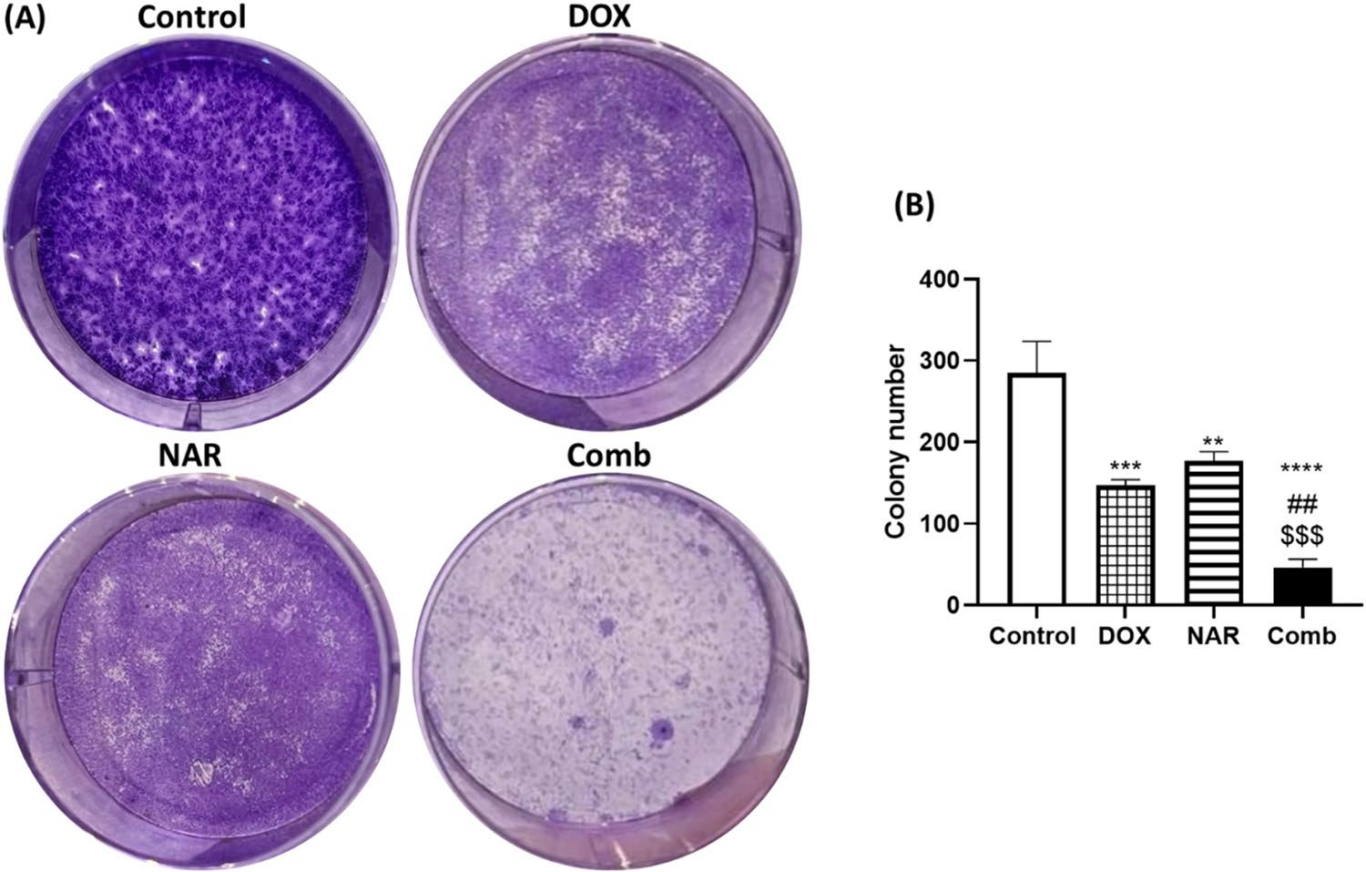
Effect of doxorubicin and naringin on colony formation. (**A**) Representative images of MCF-7 cells without or with DOX and NAR treatment then stained with crystal violet. (**B**): Colony number counted and presented as mean ± SD (n = 3). One-way ANOVA followed by post-hoc Tukey’s test was performed. *^,#,$^p˂0.05, **^,##,$$^ p˂0.01, ***^,###,$$$^ p˂0.001, ****^,####,$$$$^ p˂0.0001. *, #, $ are significant differences from control, DOX, and NAR respectively.

MCF-7 proliferation was investigated by SRB assay. The results indicated that both doxorubicin and naringin significantly reduced MCF-7 viability in a dose-dependent way (Fig. 1A,B). Doxorubicin individually inhibited MCF-7 growth with an IC_50_ of 5.7 μg/mL while naringin decreased the growth with IC_50_ of 15.3 μg/mL. Furthermore, IC_50_ of doxorubicin was decreased from 5.7 to 2.1 μg/ml upon the addition of 15.3 μg/ml naringin that could indicate their synergetic effect. Moreover, our data demonstrated that naringin has greater cytotoxicity on MCF-7 cancer cells than HSF normal cells. IC_50_ value of NAR for HSF cells could not be calculated as the value was out of range (Fig. 1C). Doxorubicin and naringin were found to inhibit MCF-7 cell growth, this inhibition was then confirmed using colony formation assay. As seen in Fig. 2, the colony numbers were remarkably decreased in MCF-7 cells treated with DOX (*p*˂0.001), NAR (*p*˂0.01), or their combination (*p*˂0.0001) relative to untreated cells. Further, the combination treatment group demonstrated lower colony numbers compared to cells treated with DOX or NAR alone.

### Doxorubicin and naringin combination induce downregulation of JAK1/STAT3 signaling and their downstream targets

**Figure 3:**
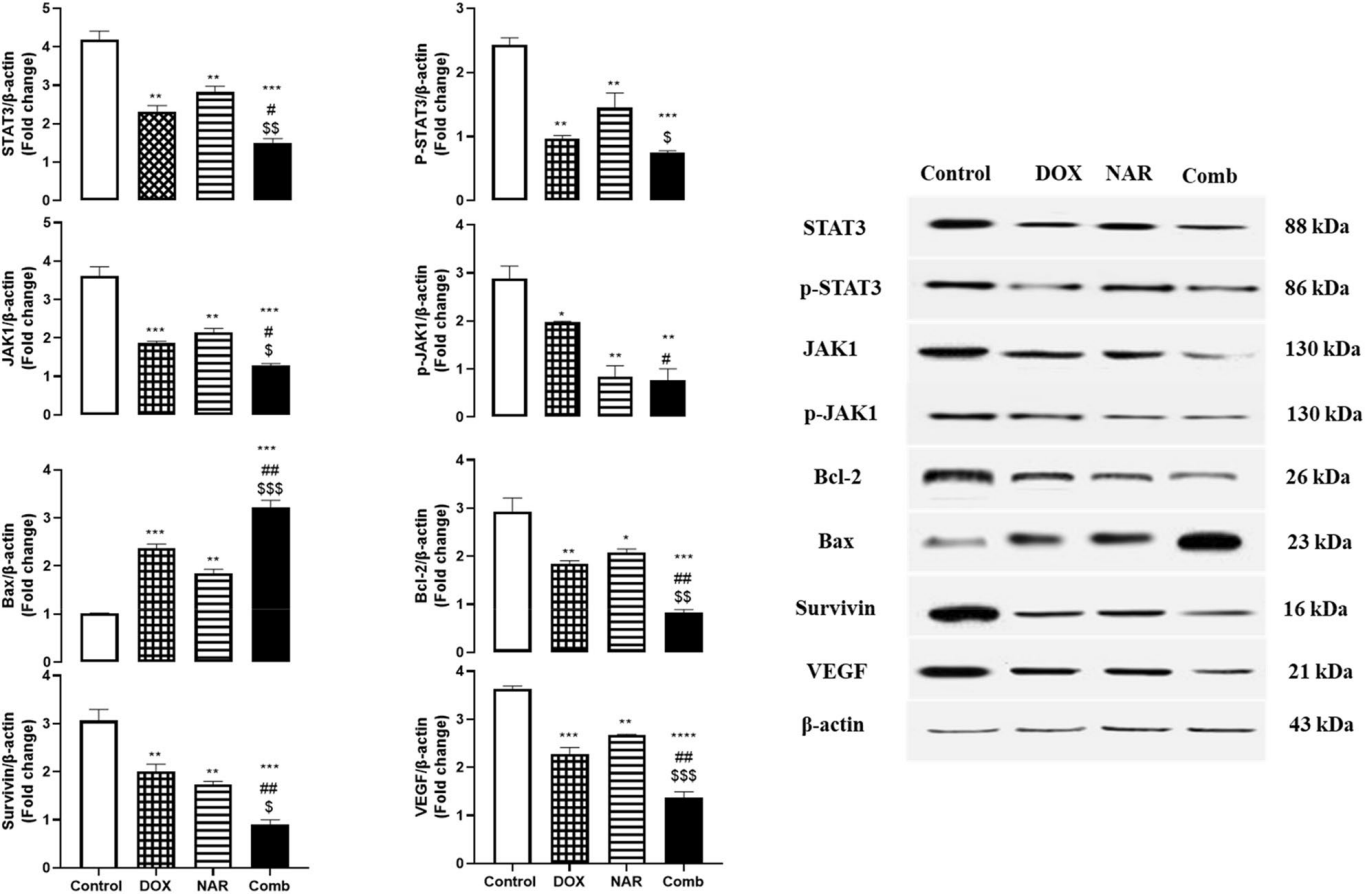
Impact of doxorubicin and naringin alone or in combination on expression level of STAT3, JAK1, Bax, Bcl-2, Survivin, and VEGF proteins in MCF-7 cells. The cells were treated with ½ IC_50_ DOX, IC_50_ NAR, and combination of both for 48h. By western blot technique, the target proteins were detected and normalized to β-actin then quantified by image J software. The data are presented as mean ± SD (n = 3). One-way ANOVA followed by post-hoc Tukey’s test was performed. *^,#,$^p˂0.05, **^,##,$$^ p˂0.01, ***^,###,$$$^ p˂0.001, ****^,####,$$$$^ p˂0.0001. *, #, $ are significant differences from control, DOX, and NAR respectively.

**Figure 4:**
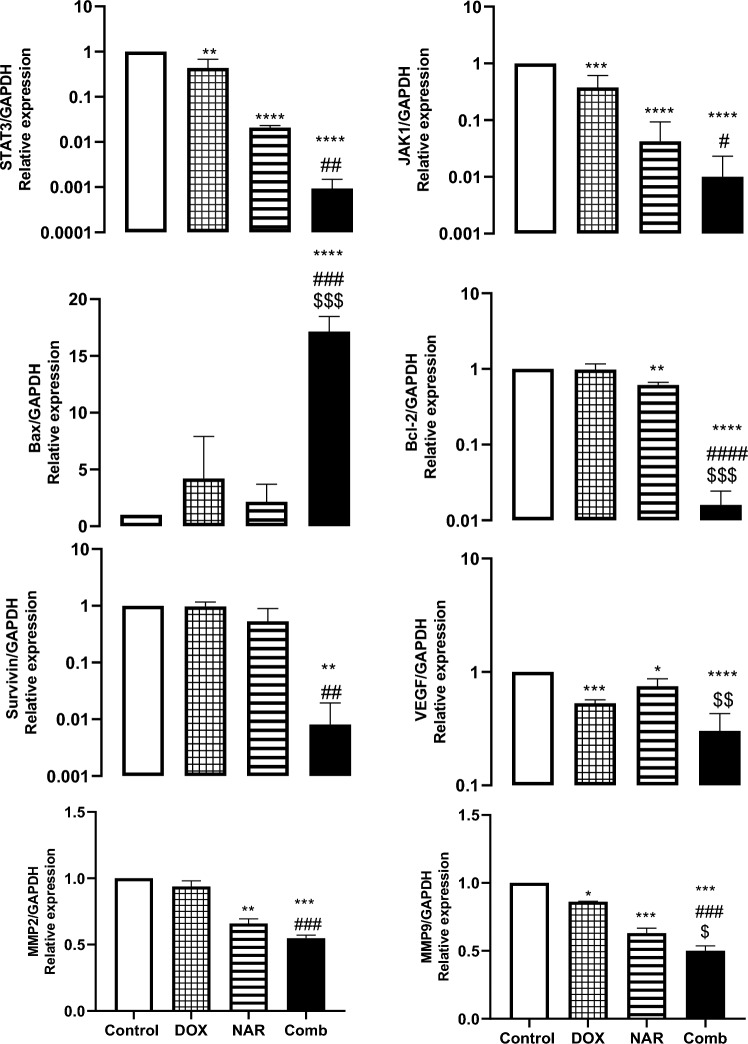
The relative expression of STAT3, JAK1, Bax, Bcl-2, Survivin, VEGF, MMP2, and MMP9 genes in MCF-7 breast cancer cells using qPCR. The cells were treated in vitro using ½ IC_50_ DOX, IC_50_ NAR, and combination of both for 48h. The data are presented as mean ± SD (n = 3). One-way ANOVA followed by post-hoc Tukey’s test was performed. *^,#,$^p˂0.05, **^,##,$$^ p˂0.01, ***^,###,$$$^ p˂0.001, ****^,####,$$$$^ p˂0.0001. *, #, $ are significant differences from control, DOX, and NAR respectively.

Western blot study of MCF-7 cells treated with DOX, NAR, or their combination revealed significant downregulation in the expression of STAT3, p-STAT3, JAK1, and p-JAK1 48h later, as shown in Fig. 3. In addition, the mRNA expression level of STAT3 and JAK1 was dramatically decreased in MCF-7 cells treated with either DOX, NAR or their combination as quantified using qRT-PCR (Fig. 4).

We then further examined the expression of different apoptosis, cell growth, and invasion regulatory proteins known to be STAT3 pathway downstream targets. At 48h, DOX, NAR, or both stimulated downregulation of Bcl-2 concurrently with upregulation of Bax in MCF-7 cells (Fig. 3). Moreover, a considerable reduction in the expression of survivin and VEGF proteins was observed in MCF-7 cells treated with either DOX or NAR. The combination treatment showed the lowest expression compared to single therapy. According to qRT-PCR data in Fig. 4, no significant change in Bax and survivin mRNA expression level was detected in MCF-7 cells treated with DOX or NAR. The combinational treatment, on the other hand, demonstrated potent down regulation of Bcl-2, and survivin levels with notable increase in Bax expression (Fig. 4). The obtained results elucidated that the combined treatment may inhibit proliferation and induce apoptosis in breast cancer via suppressing JAK/STAT signaling pathway. Further, the mRNA expression levels of MMP-2 and MMP-9 were evaluated using qPCR. As seen in Fig. 4, MMP-2 expression level was significantly downregulated in MCF-7 cells treated with naringin alone or in combination with doxorubicin. In addition, a remarkable reduction in MMP-9 levels was observed in MCF-7 cells treated either with naringin or doxorubicin or combination of both. These obtained data figured out the possible antimetastatic effect of NAR/DOX combination.

### Doxorubicin, naringin, and their combination reduce MCF-7 cells migration

**Figure 5:**
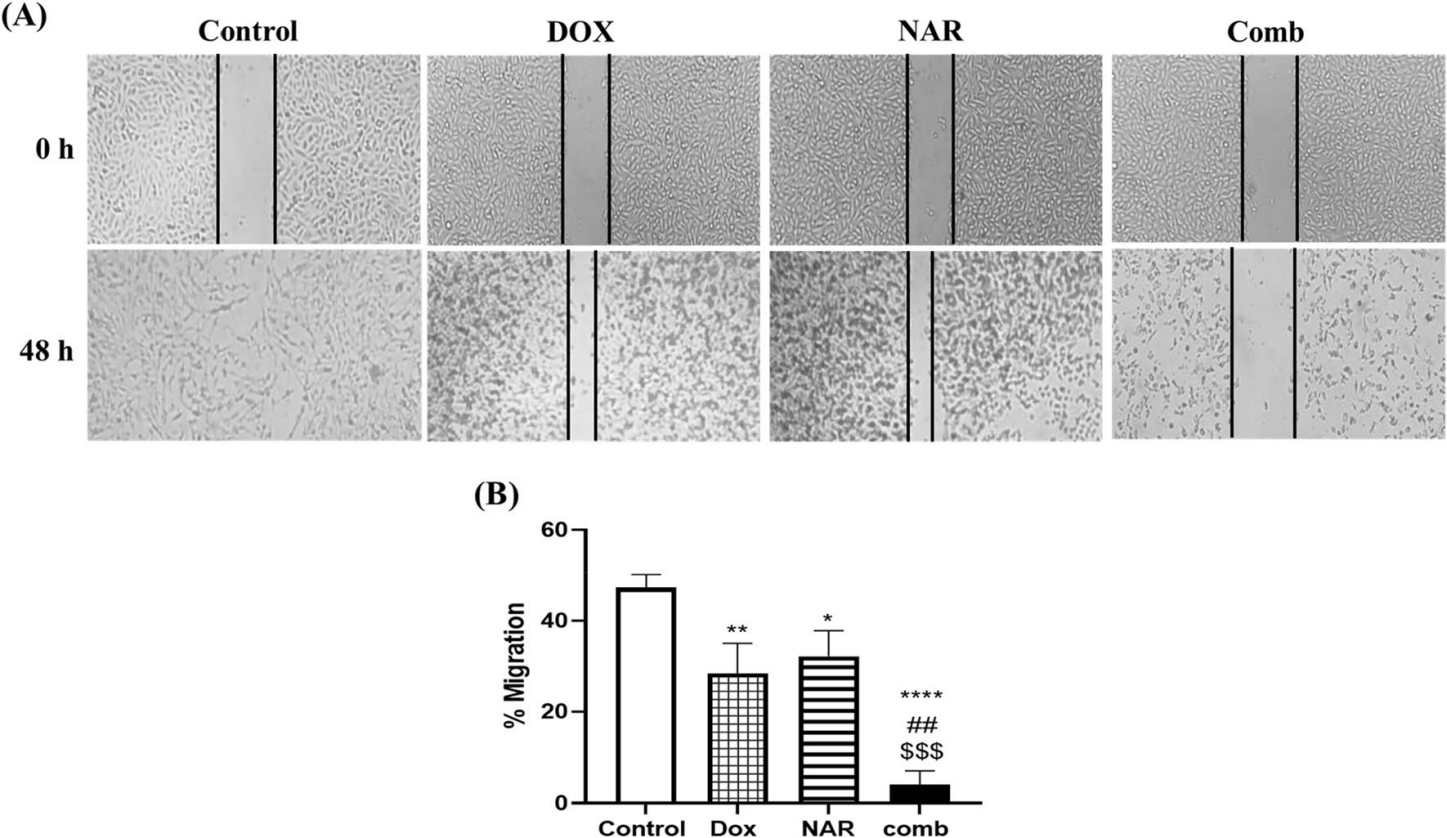
Effect of doxorubicin and naringin combination on the migration of MCF-7 cells. **A**: MCF-7 cells received ½ IC_50_ DOX, IC_50_ NAR, and combination of both and a scratch was made to assess their effect on the cell movement over 48h period. **B**: Quantitative statistical graph of scratch assay. The data are presented as mean ± SD (n = 3). One-way ANOVA followed by post-hoc Tukey’s test was performed. *^,#,$^p˂0.05, **^,##,$$^ p˂0.01, ***^,###,$$$^ p˂0.001, ****^,####,$$$$^ p˂0.0001. *, #, $ are significant differences from control, DOX, and NAR respectively.

The wound healing assay was conducted to study the effect of doxorubicin and/or naringin on the migratory ability of MCF-7 cells. In the co-presence of doxorubicin and naringin, the closure of cell-free areas was considerably diminished after 48h (Fig. 5A), and the percentage of cell migration significantly decreased from 47.27 to 4.02%. Single therapy with doxorubicin or naringin, in contrast, resulted in a remarkable decline in cell migration percentage from 47.27 to 28.39 and 32.18%, respectively, as shown in Fig. 5B. The experiment faced a limitation wherein cells treated with DOX, NAR, or their combination appeared dead after 48 h, potentially interfering with elucidating the drugs' antimigratory effect. To address this, we plan to further investigate the combined therapy's impact on breast cancer cell migration, aiming to enhance our understanding of the antimigratory effects of the drugs.

### Doxorubicin and naringin combination induce apoptosis in MCF-7 cells

**Figure 6:**
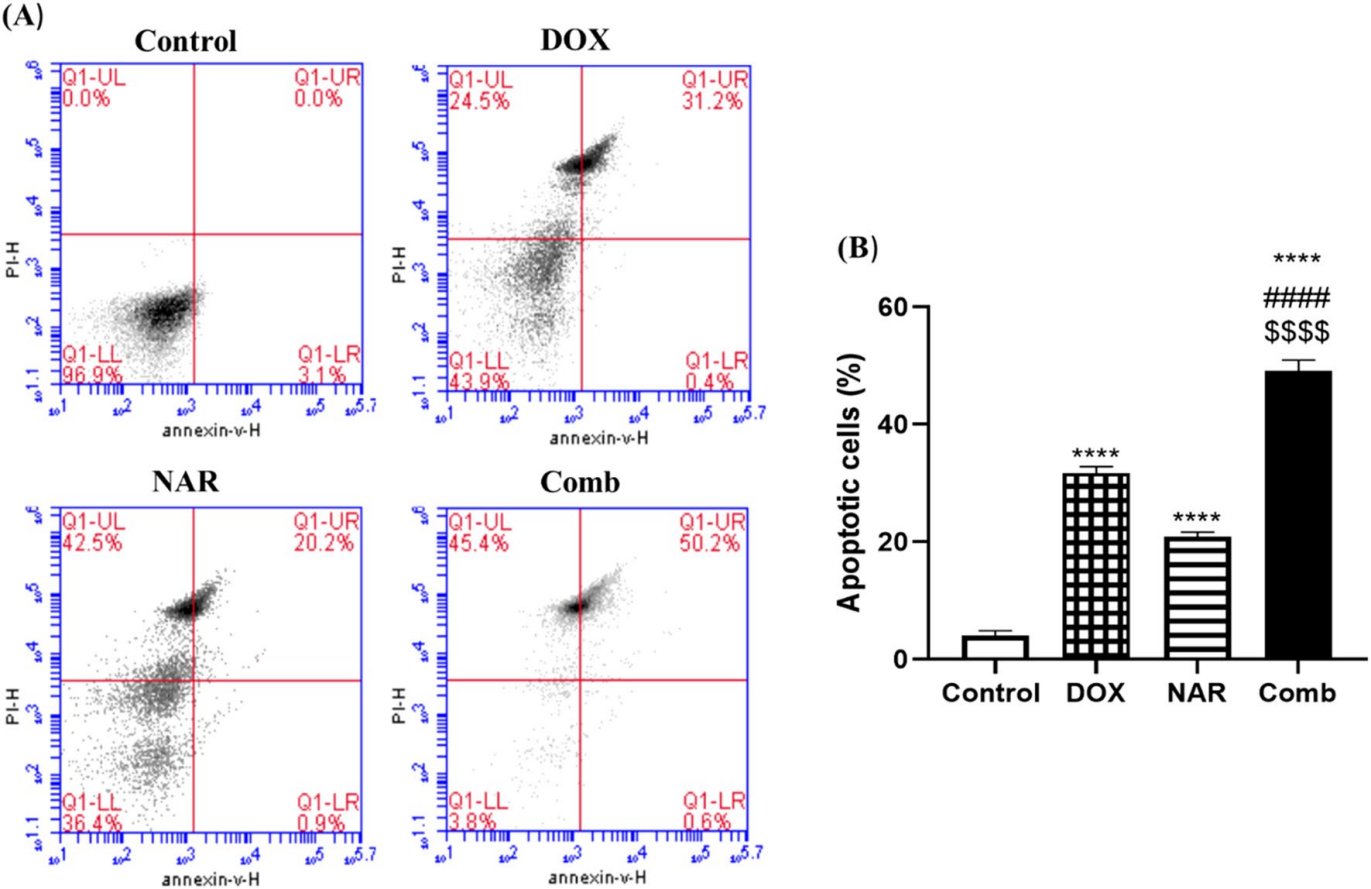
Doxorubicin and naringin combination induce apoptosis in MCF-7 cells. (**A**) Representative histogram of Annexin V and PI staining of cells treated with ½ IC_50_ DOX, IC_50_ NAR, and combination of both for 48h. (**B**) Quantitative analysis of the percentage of apoptotic cells. The data are presented as mean ± SD (n = 3). One-way ANOVA followed by post-hoc Tukey’s test was performed. *^,#,$^p˂0.05, **^,##,$$^ p˂0.01, ***^,###,$$$^ p˂0.001, ****^,####,$$$$^ p˂0.0001. *, #, $ are significant differences from control, DOX, and NAR respectively.

Because of growth inhibitory effects mentioned above, we further determined if DOX, NAR or their combination can induce apoptosis in MCF-7 cells. As depicted in Fig. 6, flow cytometric analysis demonstrated that the percentage of apoptotic cells increased from 3.1 to 50.8% after the treatment of MCF-7 cells with doxorubicin and naringin combination. Additionally, DOX or NAR treatment caused a statistically significant (*p*˂0.0001) increase in the percentage of apoptotic cells to 31.6 and 21.2% respectively. On the other hand, the percentage of viable cells decreased from 96.9 to 3.8% after 48h treatment with the combined therapy.

### Naringin directly interacts with STAT3 by docking interaction analysis

**Figure 7:**
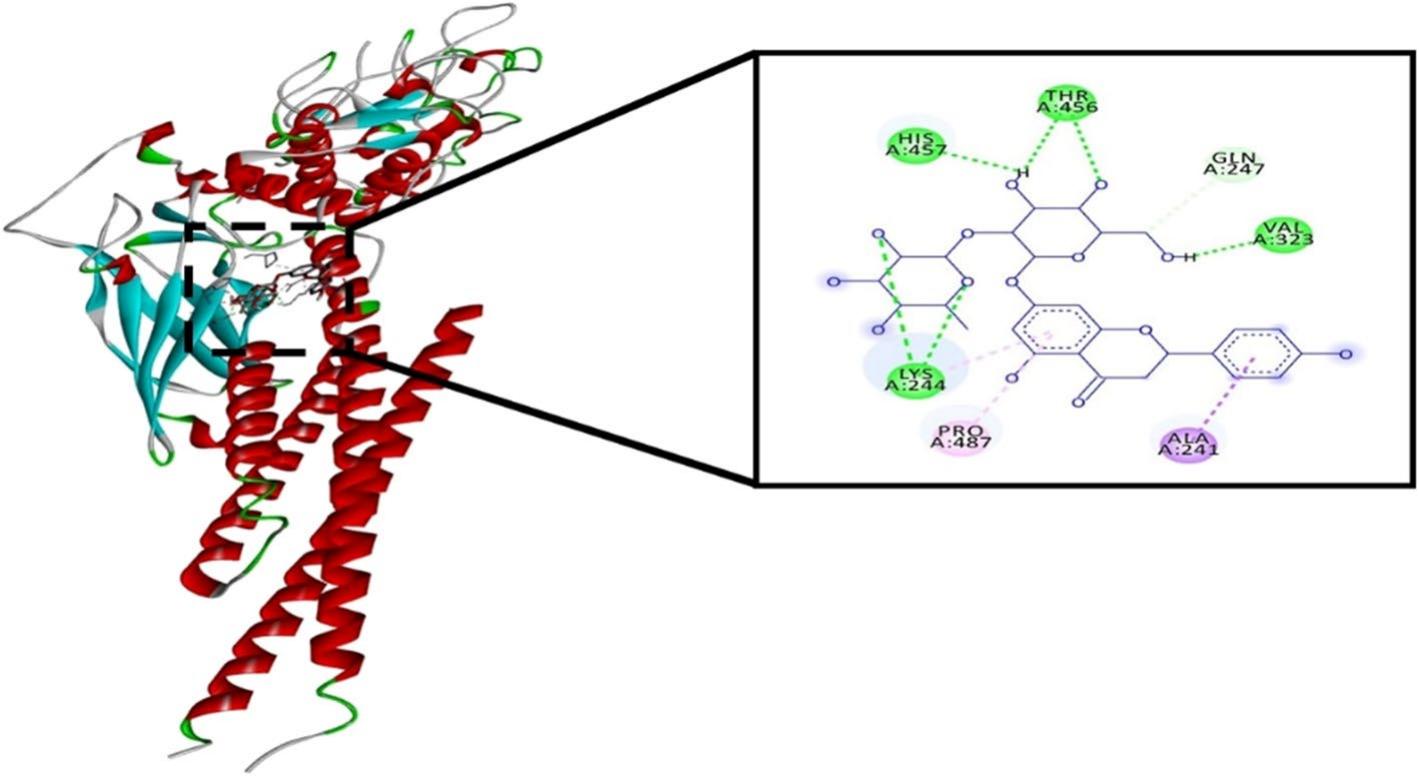
Naringin's predicted mechanism of interaction with the STAT3 protein, as demonstrated by computational docking. Structure of STAT3 is demonstrated as ribbon surface model. Naringin shows interactions with residues Lys244, Thr456, and His457 of STAT3 as shown in the expanded panel. The hydrophobic interactions and hydrogen bonds are shown as dashed-purple and green lines respectively.

The results of the molecular docking demonstrated that naringin and STAT3 protein could bind and interact together with estimated binding energy of -8.02 kcal/mol, and inhibition constant (Ki) of 1.33 µM. Figure 7 shows DSV analysis of the best docking complex. As shown in this figure, naringin formed 5 hydrogen bonds with Lys244, His457, and Thr 456 and 3 hydrophobic interactions with Lys244, Ala241, and Pro487.

## Discussion

Currently, cancer treatment strategies priorities a combination of natural products and chemically synthesized drugs such as doxorubicin to increase their effectiveness and minimize side effects^29^. Thus, the research's primary aim is to examine if naringin, alone or in combination with the chemotherapeutic drug doxorubicin, could inhibit the growth and multiplication of MCF-7 breast cancer cells by suppressing the STAT3 signaling pathway. We show that naringin and doxorubicin individually retarded the viability of MCF-7 after 48 h with IC_50_ of 15.3 and 5.7 µg/mL respectively. Further, the combination of different doxorubicin doses with IC_50_ of naringin produced maximum cytotoxicity compared to independent compound treatment, indicating synergistic source of new and efficient anticancer drugs as previously described^30,31^.

Phosphorylation of JAK and STAT is a crucial step in tumor cell proliferation and transformation. As a result, inhibiting STAT3 activation and phosphorylation prevents its dimerization and DNA binding. So, downregulation of STAT3-regulated gene expression (Bcl-2, survivin, and VEGF) inhibits cancer cell proliferation and induces apoptosis^32^. Bcl-2 is antiapoptotic protein that inhibit tumor cell apoptosis whereas Bax is pro-apoptotic protein^33^. Survivin is also apoptosis inhibitor and modulate cell development and proliferation^34^. VEGF is angiogenic factor that promote cancer invasion and metastasis^35^. As expected, in the current work, MCF-7 cells incubated with a combination of naringin and doxorubicin, revealed a substantial downregulation in the expression of JAK1, STAT3, Bcl-2, survivin, and VEGF genes, as well as a remarkable upregulation in the expression of Bax. Moreover, immunoblotting displayed that naringin and doxorubicin combinational therapy inhibit JAK1 and STAT3 signaling pathway. These results were confirmed using computational modeling that demonstrated the mode of interaction of naringin with amino acids Lys244, His457, Pro487, and Thr 456. These findings suggested that naringin may inhibit JAK/STAT signaling pathway through its interaction with DNA binding domain (DBD) of STAT3. Like other STAT family, STAT3 protein is consist of 6 different motifs, including Amino-terminal domain (NTD), coiled-coil domain, DNA-binding domain, linker domain (LD), Src Homology 2 (SH2) domain and trans-activation domain (TAD). Each motif plays an essential role in STAT3 phosphorylation and dimerization. In the current work, naringin showed interaction with DBD domain which important for STAT3 dimer binding with specific DNA sequence in the nucleus and enhance gene expression^36,37^. Our findings support prior study that found garcinol suppressed the phosphorylation of STAT3. As a result, STAT3-regulated genes Bcl-2, cyclin D1, Bcl-xL, survivin, Mcl-1, and VEGF were downregulated, inhibiting proliferation and prompting substantial death in hepatocellular carcinoma cells^38^.

Metastasis is one of the most frequent reasons of chemoresistance and treatment failure in breast cancer^39^. The migration assay revealed that naringin treatment alone or combined with doxorubicin considerably reduced the capacity of MCF-7 cells to spread. The antimetastatic activities of naringin were explained by a drop in the levels of STAT3-regulated gene products implicated in cell invasion (e.g., VEGF). Previous studies suggested that STAT3 activation play a crucial role in the cancer invasion and metastasis by modulating the MMPs expression^40–42^.Our findings revealed that the expression level of MMP2 and MMP9 was remarkably reduced in MCF-7 cells treated with NAR and DOX. These observations are comparable with those of Zhu et al. who found that naringin could significantly reduce the migration and invasion of MGC803 and MKN45 gastric cancer cells by transwell and scratch assay^43^. All the evidence suggested that naringin and doxorubicin combination therapy may be more effective than naringin and doxorubicin alone at slowing cell propagation, stimulating apoptosis, and lowering migration in breast cancer cells.

## Conclusion

These findings indicate that combinational therapy can be a more efficient drug than the two compounds, naringin and doxorubicin, taken separately. Doxorubicin combined with naringin may be investigated as a treatment alternative for breast cancer. Further in vivo studies could be conducted to validate the safety of this combination.

### Supplementary Information

Supplementary Information.

## Data Availability

All the data investigated and shown in this work is accessible from the authors upon an appropriate request.

## Abbreviations

DOX: Doxorubicin
Bcl-2: B-cell lymphoma 2
Bax: BCL2 associated X
VEGF: Vascular endothelial growth factor
STAT3: Signal transducer and activator of transcription 3
JAK1: Janus kinase 1

## References

[1] Abdelaziz AH (2021). Breast cancer awareness among Egyptian women and the impact of caring for patients with breast cancer on family caregivers’ knowledge and behaviour. Res. Oncol..

[2] Wang X, Zhang H, Chen X (2019). Drug resistance and combating drug resistance in cancer. CDR (Alhambra Calif.).

[3] Alfarouk KO (2015). Resistance to cancer chemotherapy: Failure in drug response from ADME to P-gp. Cancer Cell Int..

[4] Mohajeri M, Sahebkar A (2018). Protective effects of curcumin against doxorubicin-induced toxicity and resistance: A review. Crit. Rev. Oncol. Hematol..

[5] Bayat Mokhtari R (2017). Combination therapy in combating cancer. Oncotarget.

[6] Neuhouser ML (2004). Dietary flavonoids and cancer risk: Evidence from human population studies. Nutr. Cancer.

[7] Ahmad N (2022). Dietary polyphenols: Extraction, identification, bioavailability, and role for prevention and treatment of colorectal and prostate cancers. Molecules.

[8] Alam F, Mohammadin K, Shafique Z, Amjad ST, Asad MHHb (2022). Citrus flavonoids as potential therapeutic agents: A review. Phytother. Res..

[9] Khan, U. M. *et al.* Citrus genus and its waste utilization: A review on health-promoting activities and industrial application. *Evid. Based Complement. Alternat. Med.* **2021** (2021). 10.1155/2021/2488804 PMC8595006 34795782

[10] Patil VM, Masand N (2018). Anticancer potential of flavonoids: Chemistry, biological activities, and future perspectives. Stud. Nat. Prod. Chem..

[11] Zeng L (2014). Naringin inhibits growth and induces apoptosis by a mechanism dependent on reduced activation of NF-κB/COX-2-caspase-1 pathway in HeLa cervical cancer cells. Int. J. Oncol..

[12] Bailey DG, Malcolm J, Arnold O, David Spence J (1998). Grapefruit juice–drug interactions. Br. J. Clin. Pharmacol..

[13] Yoshinaga A (2016). NEU3 inhibitory effect of naringin suppresses cancer cell growth by attenuation of EGFR signaling through GM3 ganglioside accumulation. Eur. J. Pharmacol..

[14] Chen R, Qi Q-L, Wang M-T, Li Q-Y (2016). Therapeutic potential of naringin: An overview. Pharm. Biol..

[15] Camargo CA, Gomes-Marcondes MCC, Wutzki NC, Aoyama H (2012). Naringin inhibits tumor growth and reduces interleukin-6 and tumor necrosis factor α levels in rats with Walker 256 carcinosarcoma. Anticancer Res..

[16] Guo B, Zhang Y, Hui Q, Wang H, Tao K (2016). Naringin suppresses the metabolism of A375 cells by inhibiting the phosphorylation of c-Src. Tumour Biol..

[17] Li H (2013). Naringin inhibits growth potential of human triple-negative breast cancer cells by targeting β-catenin signaling pathway. Toxicol. Lett..

[18] Zhou J, Xia L, Zhang Y (2019). Naringin inhibits thyroid cancer cell proliferation and induces cell apoptosis through repressing PI3K/AKT pathway. Pathol.-Res. Pract..

[19] Wang X, Crowe PJ, Goldstein D, Yang J-L (2012). STAT3 inhibition, a novel approach to enhancing targeted therapy in human cancers. Int. J. Oncol..

[20] Hejazi II, Shahabuddin S, Bhat AR, Athar F (2019). Pharmacokinetic evaluation, molecular docking and in vitro biological evaluation of 1, 3, 4-oxadiazole derivatives as potent antioxidants and STAT3 inhibitors. J. Pharm. Anal..

[21] Escobar Z (2016). Preclinical characterization of 3β-(N-acetyl l-cysteine methyl ester)-2aβ, 3-dihydrogaliellalactone (GPA512), a prodrug of a direct STAT3 inhibitor for the treatment of prostate cancer. J. Med. Chem..

[22] Skehan P (1990). New colorimetric cytotoxicity assay for anticancer-drug screening. JNCI.

[23] Yousuf M (2022). Naringenin as a potential inhibitor of human cyclin-dependent kinase 6: Molecular and structural insights into anti-cancer therapeutics. Int. J. Biol. Macromol..

[24] Schmittgen TD, Livak KJ (2008). Analyzing real-time PCR data by the comparative CT method. Nat. Protocols.

[25] Arora A, Siddiqui IA, Shukla Y (2004). Modulation of p53 in 7, 12-dimethylbenz [a] anthracene–induced skin tumors by diallyl sulfide in Swiss albino mice. Mol. Cancer Ther..

[26] Abosharaf HA, Diab T, Atlam FM, Mohamed TM (2020). Osthole extracted from a citrus fruit that affects apoptosis on A549 cell line by histone deacetylasese inhibition (HDACs). Biotechnol. Rep..

[27] Swaminathan, G. *et al.* Molecular docking investigation of anti-viral action of Illicium Verum (star anise) against Marburg virus through Biovia discovery studio visualizer 21.1. 0.0.

[28] Yahaya MAF (2021). Insights from molecular docking and molecular dynamics on the potential of vitexin as an antagonist candidate against lipopolysaccharide (LPS) for microglial activation in neuroinflammation. BMC Biotechnol..

[29] Sundaram, M. K. *et al.* Combinational use of phytochemicals and chemotherapeutic drugs enhance their therapeutic potential on human cervical cancer cells. *Int. J. Cancer Manag.* **12** (2019).

[30] Arumugam P (2021). Synergistic effect of anethole and doxorubicin alleviates cell proliferation, cell cycle arrest, and ER stress and promotes ROS-mediated apoptosis in triple-negative breast cancer cells. J. Biochem. Mol. Toxicol..

[31] Frión-Herrera Y, Gabbia D, Carrara M (2018). Combination treatment of cuban propolis and nemorosone with chemotherapeutic agents induce a synergisitic cytotoxic effect in drug-resistant human colon carcinoma cells. J. Apitherapy Nat..

[32] Bose S (2020). Targeting the JAK/STAT signaling pathway using phytocompounds for cancer prevention and therapy. Cells.

[33] Del Principe MI (2016). Clinical significance of bax/bcl-2 ratio in chronic lymphocytic leukemia. Haematologica.

[34] Mitrović Z (2011). Prognostic significance of survivin and caspase-3 immunohistochemical expression in patients with diffuse large B-cell lymphoma treated with rituximab and CHOP. Pathol. Oncol. Res..

[35] Duffy, A. M., Bouchier-Hayes, D. J. & Harmey, J. H. in *Madame Curie bioscience database [Internet]* (Landes Bioscience, 2013).

[36] Sgrignani J (2018). Structural biology of STAT3 and its implications for anticancer therapies development. Int. J. Mol. Sci..

[37] La Sala G (2020). Selective inhibition of STAT3 signaling using monobodies targeting the coiled-coil and N-terminal domains. Nat. Commun..

[38] Sethi G (2014). Inhibition of STAT3 dimerization and acetylation by garcinol suppresses the growth of human hepatocellular carcinoma in vitro and in vivo. Mol. Cancer.

[39] Dattachoudhury S, Sharma R, Kumar A, Jaganathan BG (2020). Sorafenib inhibits proliferation, migration and invasion of breast cancer cells. Oncology.

[40] Momtaz S (2017). STAT3 targeting by polyphenols: Novel therapeutic strategy for melanoma. Biofactors.

[41] Li HD (2011). STAT3 knockdown reduces pancreatic cancer cell invasiveness and matrix metalloproteinase-7 expression in nude mice. PLoS One.

[42] Xie T-X (2004). Stat3 activation regulates the expression of matrix metalloproteinase-2 and tumor invasion and metastasis. Oncogene.

[43] Zhu L (2023). Naringin inhibits the proliferation, migration, invasion and epithelial-to-mesenchymal transition of gastric cancer cells via the PI3K/AKT signaling pathway. Alternat. Therap. Health Med..

