# Supplementary material for: Combined effects of naringin and doxorubicin on the JAK/STAT signaling pathway reduce the development and spread of breast cancer cells
Source: Sci Rep. 2024 Feb 3;14:2824. doi: 10.1038/s41598-024-53320-9 (PMC10838285; doi:10.1038/s41598-024-53320-9)
Supplement: Supplementary file 1 — Supplementary Information. [file 41598_2024_53320_MOESM1_ESM.pdf]

Supplementary materials

Combined effects of naringin and doxorubicin on the JAK/STAT signaling pathway reduce the development and spread of breast cancer cells.

Heba Effat[1*], Hamed A. Abosharaf[2], Aliaa M. Radwan[2*]

[1]Medical Biochemistry and Molecular Biology Unit, Department of Cancer Biology, National Cancer Institute, Cairo University, Cairo, Egypt. hebatullah.effat@nci.cu.edu.eg

[2]Biochemistry Division, Chemistry Department, Faculty of Science, Tanta University, Tanta, Egypt. Hamed_biochemistry@science.tanta.edu.eg
alyaa_radwan@science.tanta.edu.eg

[*]Correspondence to:
Aliaa M. Radwan
alyaa_radwan@science.tanta.edu.eg
Heba Effat
hebatullah.effat@nci.cu.edu.eg

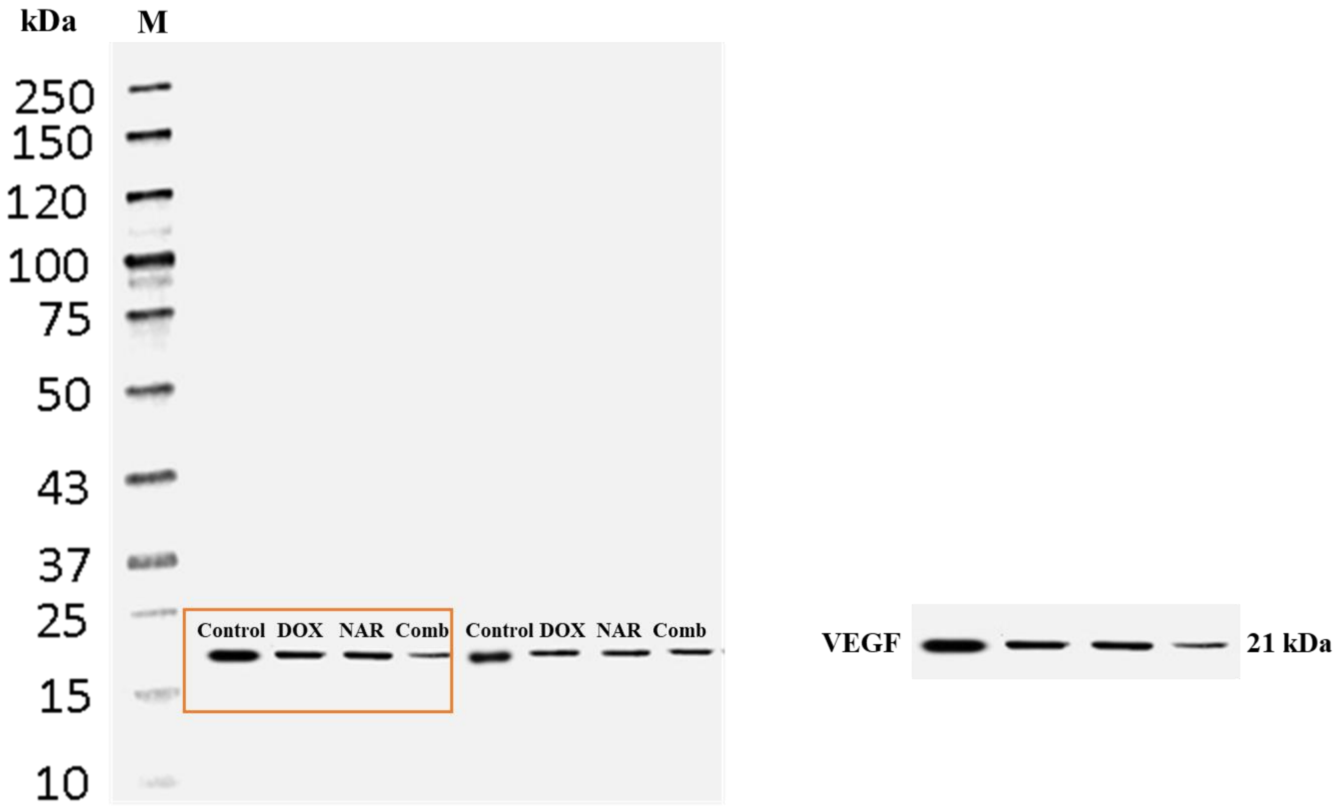

Figure S1: Original western blot image of VEGF protein

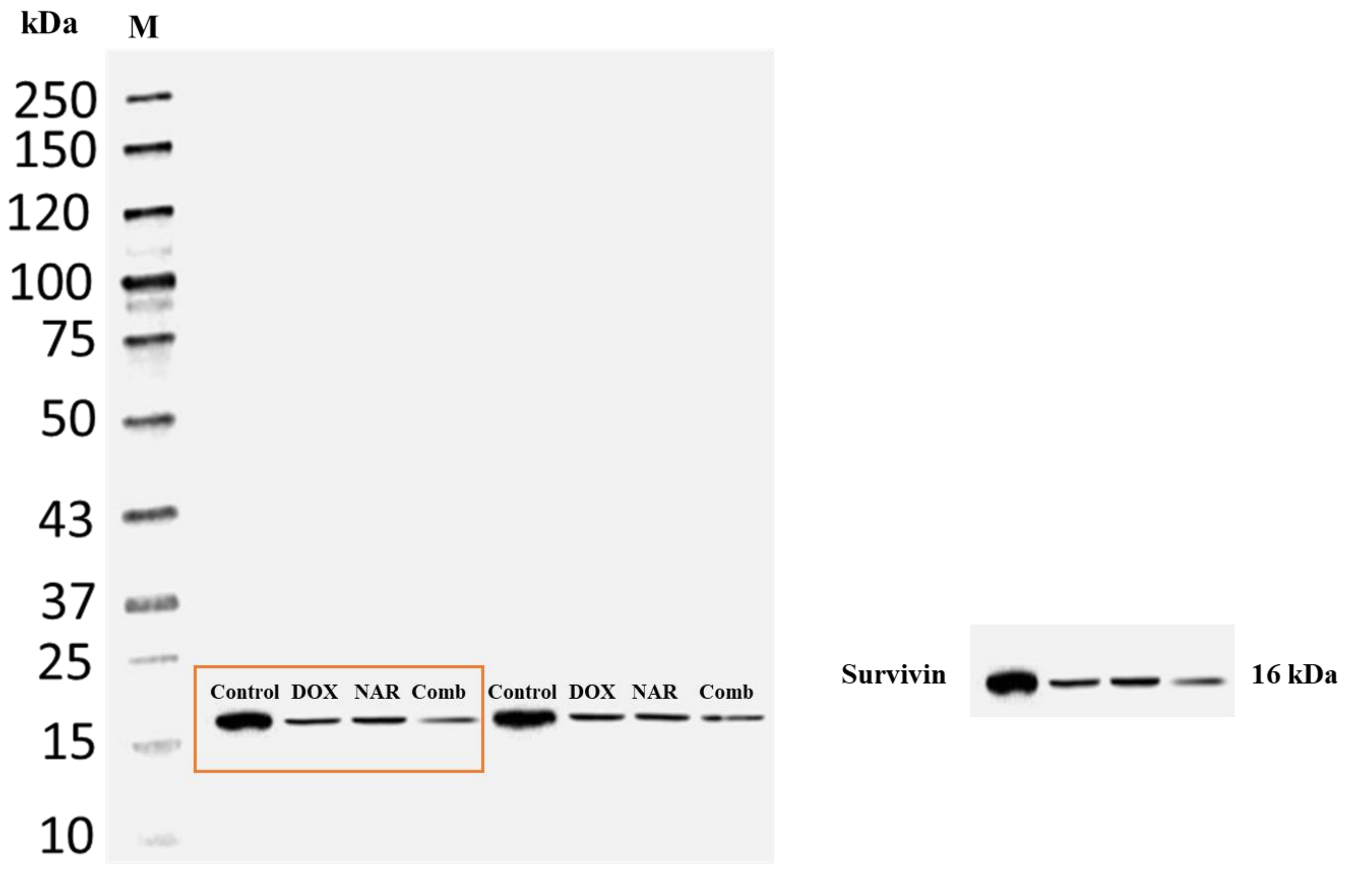

Figure S2: Original western blot image of survivin protein

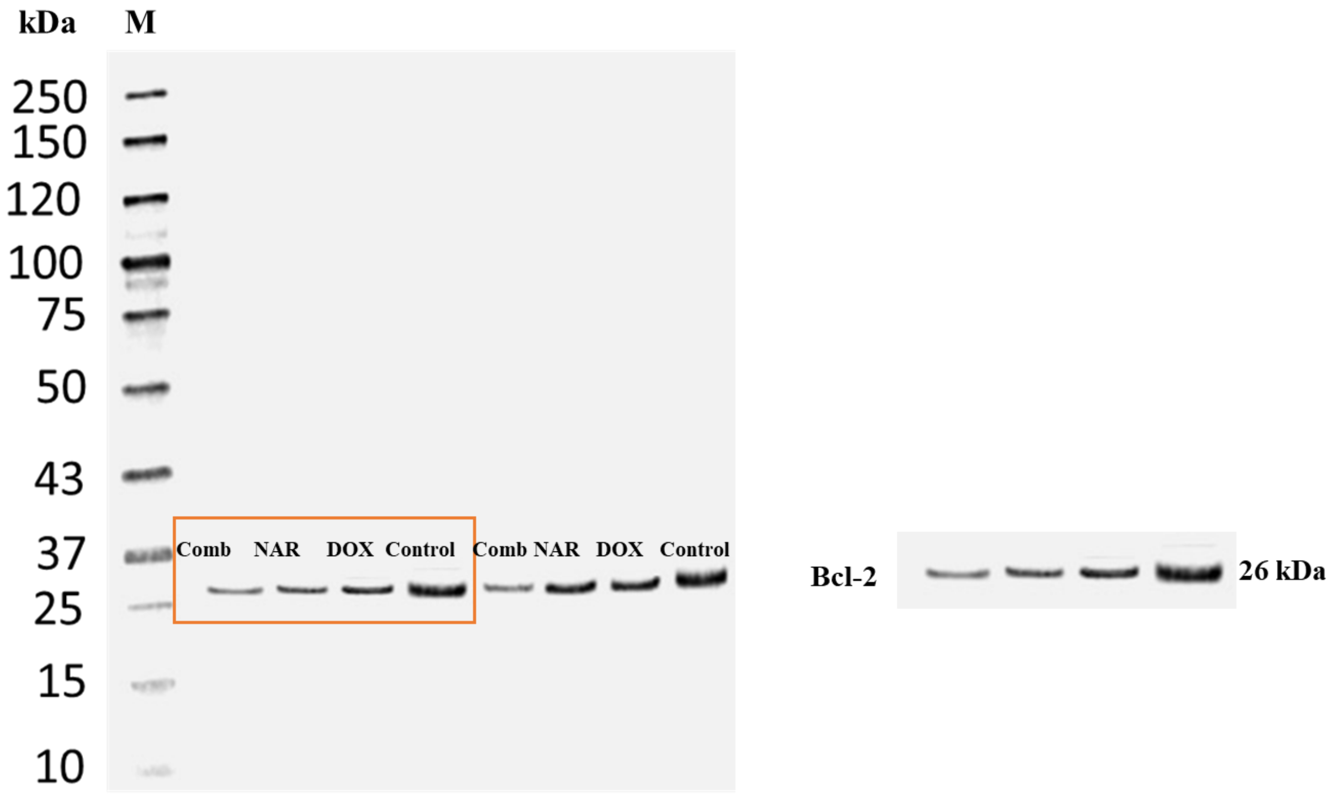

Figure S3: Original western blot image of Bcl-2 protein

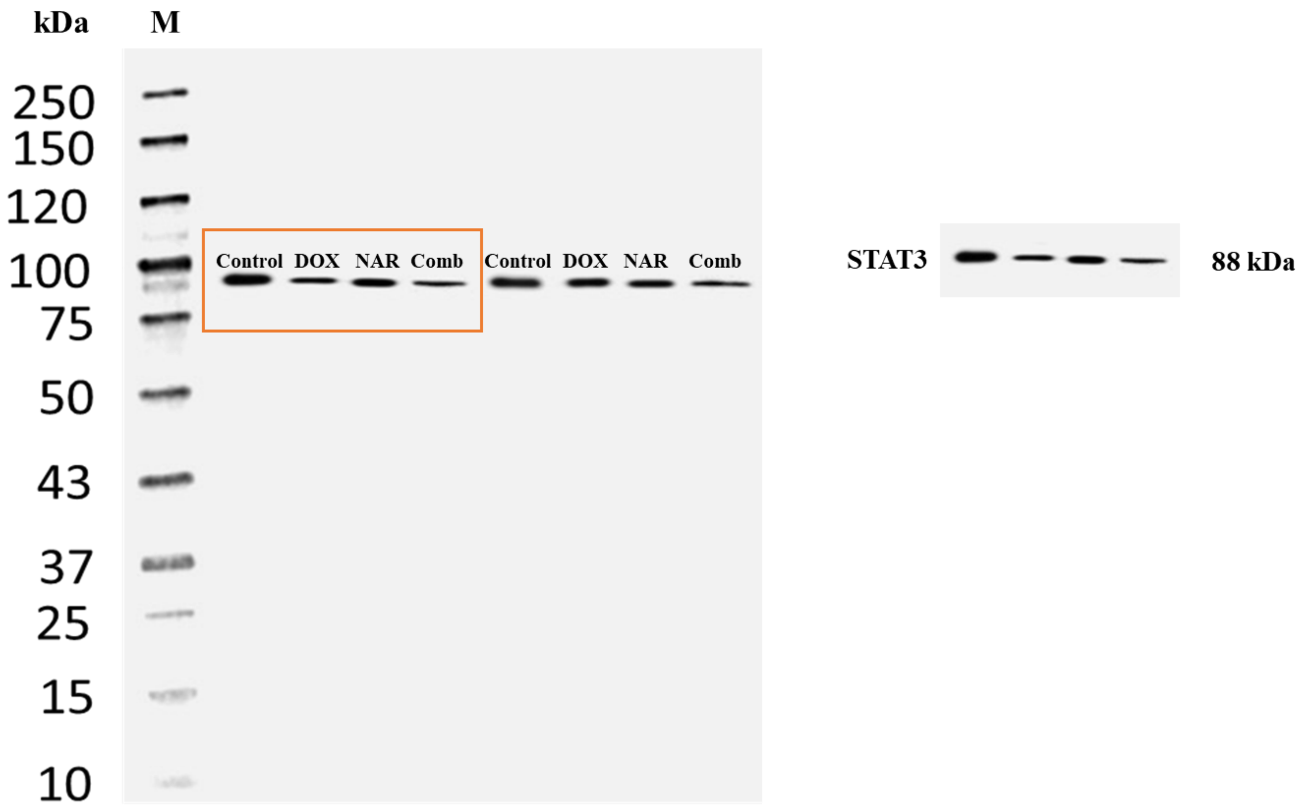

Figure S4: Original western blot image of STAT3

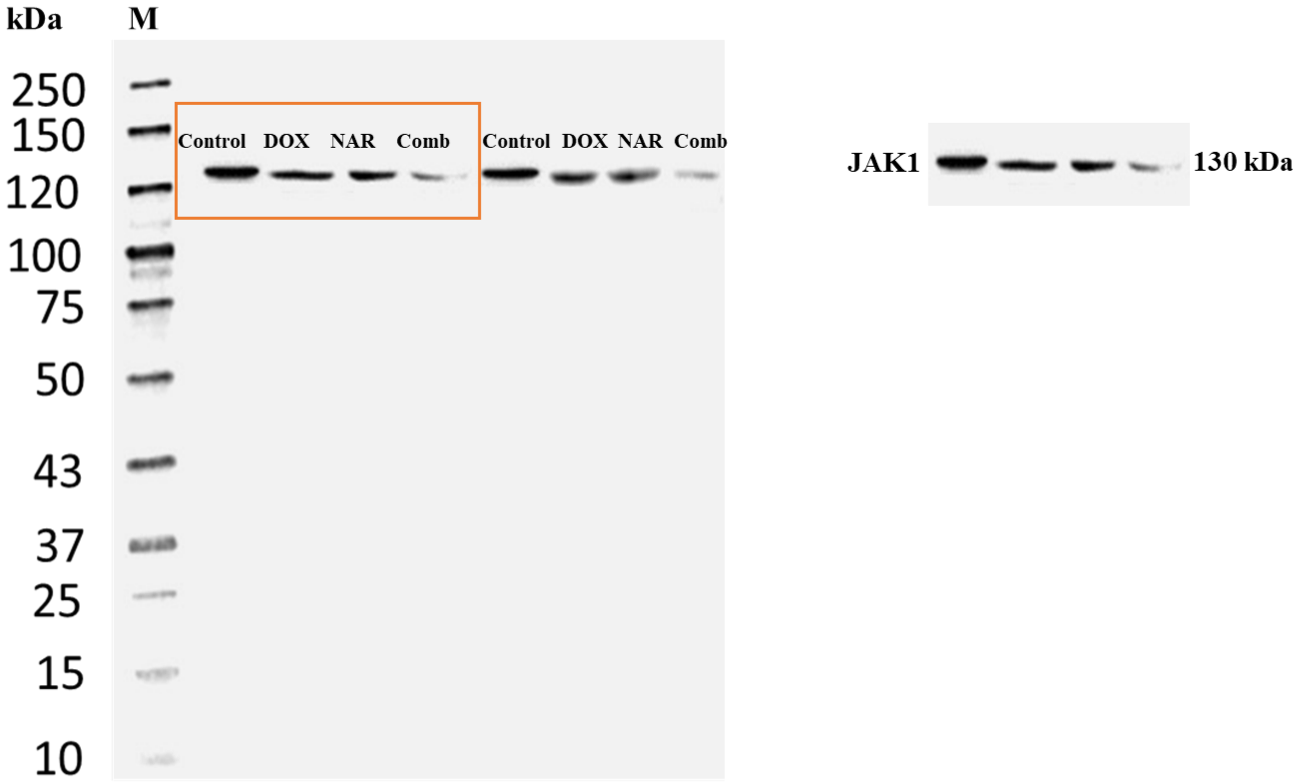

Figure S5: Original Western blot image of JAK1

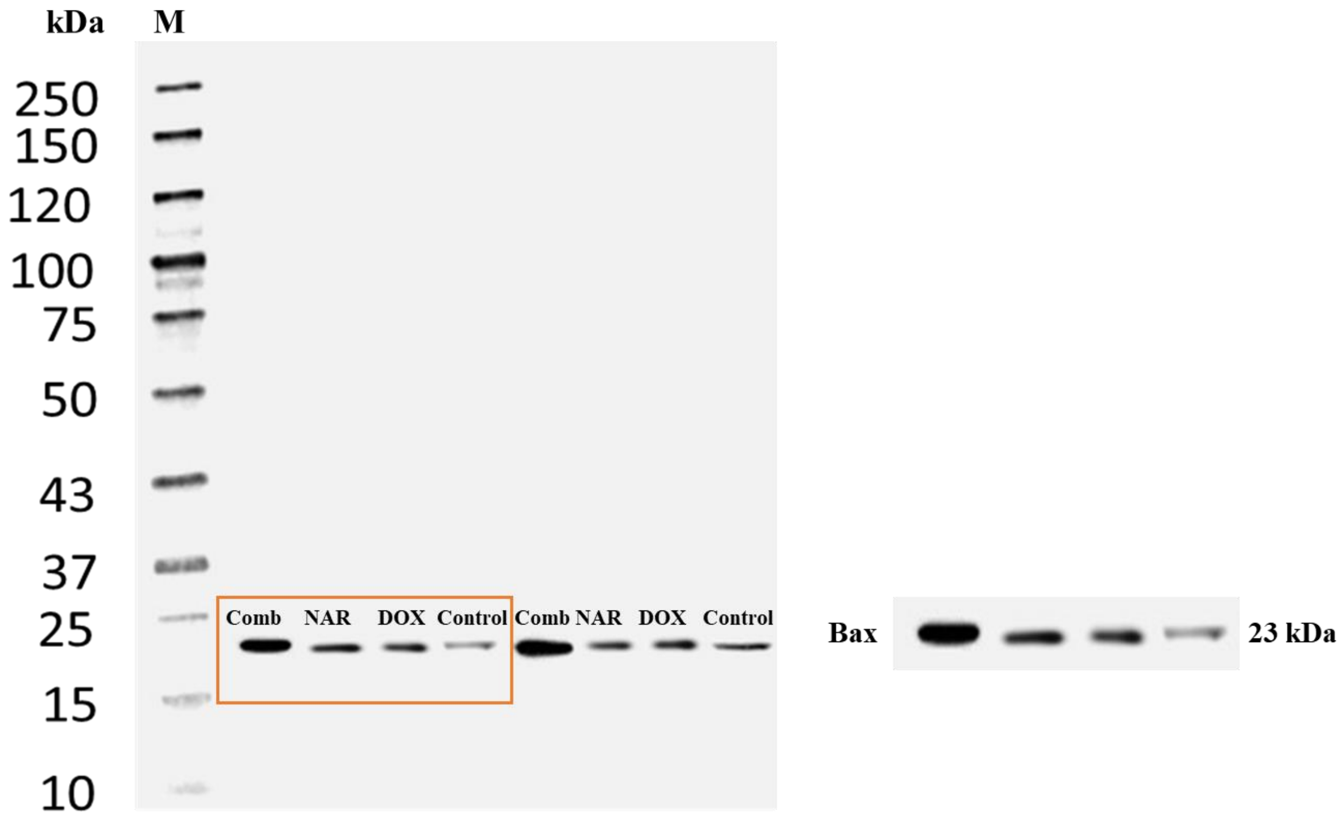

Figure S6: Original Western blot image of Bax

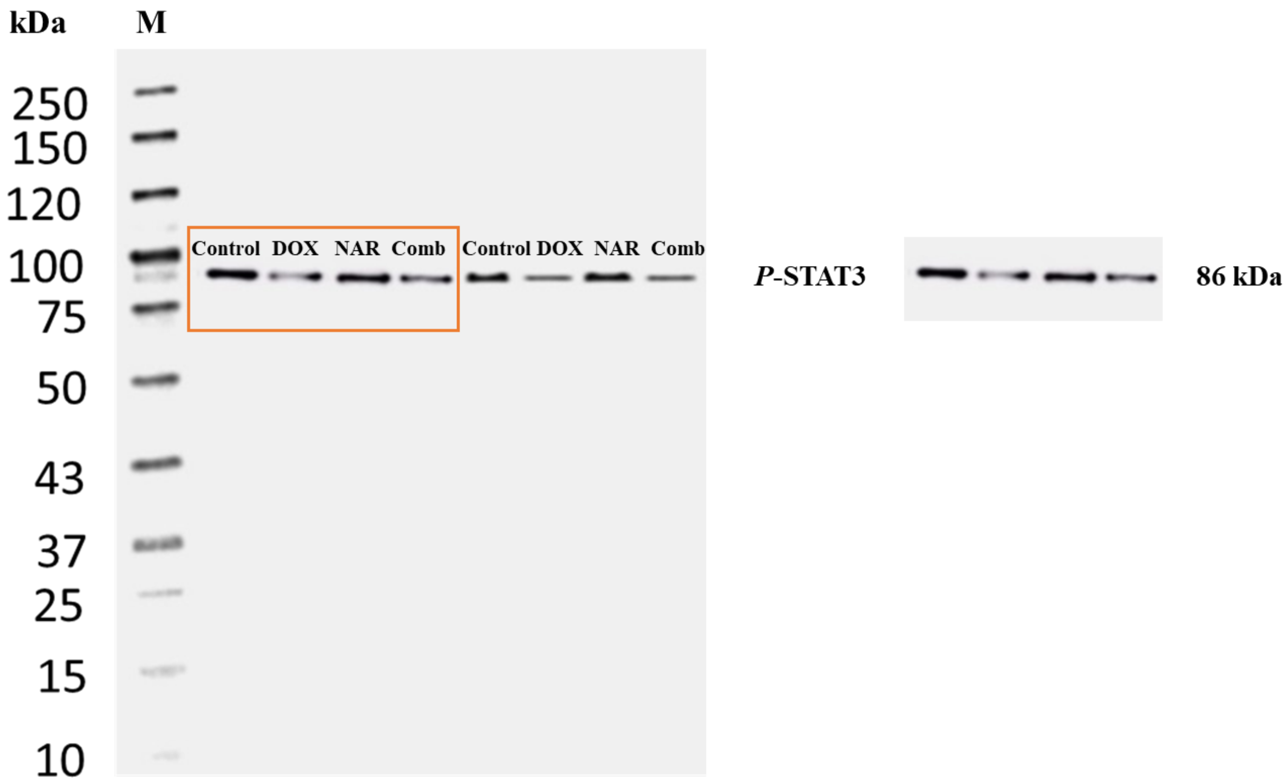

Figure S7: Original western blot image of phosphorylated STAT3

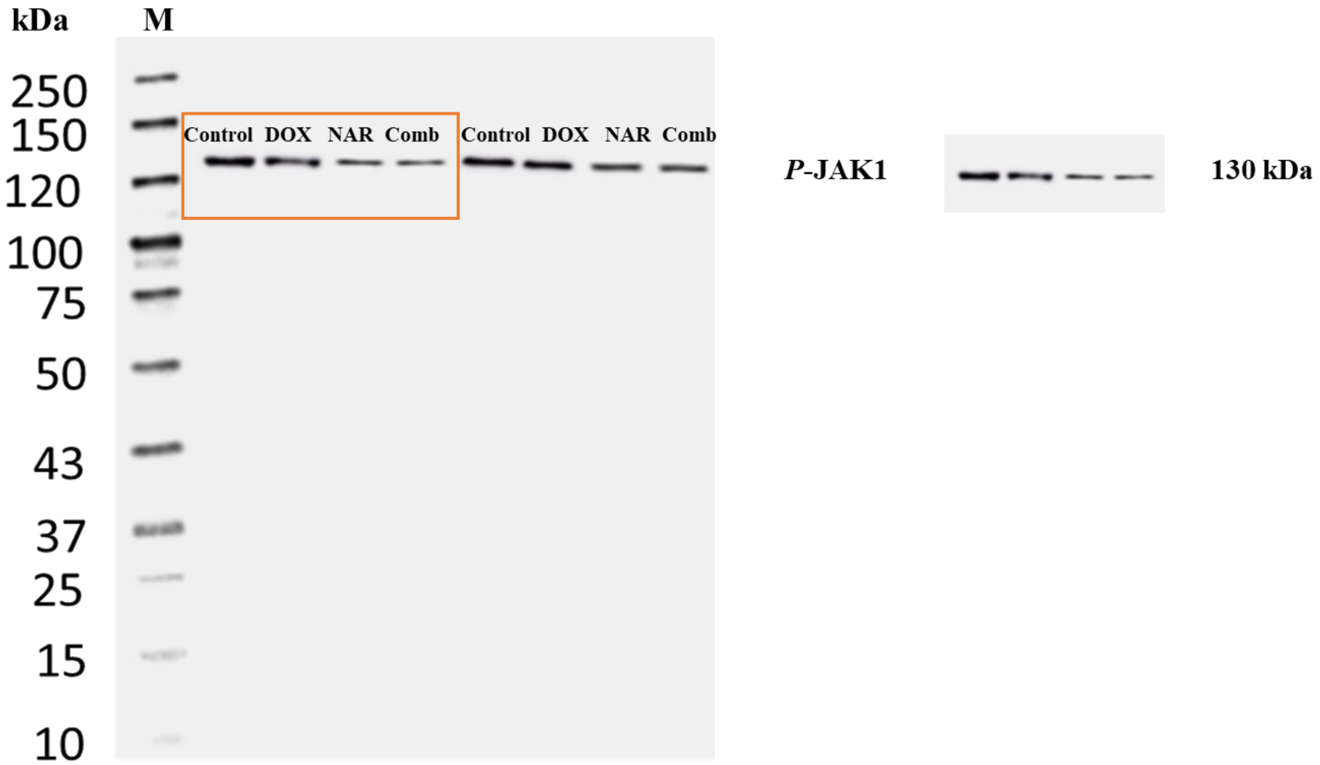

Figure S8: Original western blot image of phosphorylated JAK1

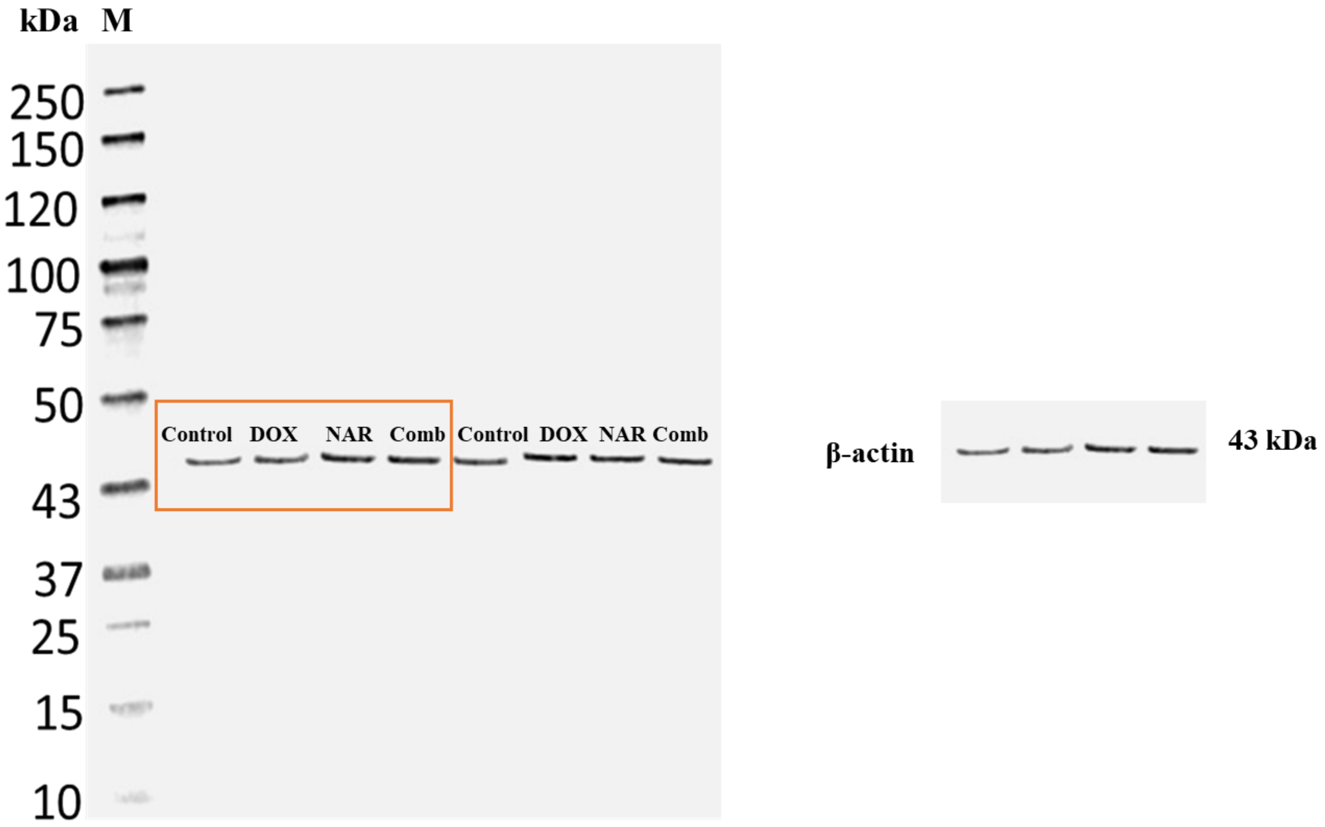

Figure S9: Original western blot image of β-actin
